# Psychiatrische Versorgung in Südtirol

**DOI:** 10.1007/s00115-024-01755-w

**Published:** 2024-10-04

**Authors:** Gerd Schaller, Roger Pycha, Andreas Conca, Tilman Steinert

**Affiliations:** 1 Psychiatrische Dienste Brixen, Brixen, Italien; 2 Psychiatrische Dienste Bozen, Bozen, Italien; 3 Zentrum für Psychiatrie Südwürttemberg (Weissenau), Klinik für Psychiatrie und Psychotherapie, Universität Ulm, Weingartshofer Str. 2, 88214 Ravensburg, Deutschland

**Keywords:** Unterbringung, Forensische Psychiatrie, Zwangsbehandlung, Gemeindepsychiatrie, Versorgungsverpflichtung, Involuntary commitment, Forensic psychiatry, Coercive treatment, Community psychiatry, Mandatory provision of care

## Abstract

Die psychiatrische Versorgung in Südtirol ist, wie überall in Italien, bis heute durch das Gesetz 180 geprägt, das 1978 unter Federführung von Franco Basaglia und Bruno Orsini in Kraft trat. Das Gesundheitsministerium legte in der Folge eine Sollbettenzahl von 10/100.000 Einwohner fest. Private Kliniken spielen in Südtirol, anders als in anderen Teilen Italiens, praktisch keine Rolle. Die „Psychiatrischen Dienste“ sind Teil des für alle Bürgerinnen und Bürger zuständigen staatlichen Gesundheitswesens und zugleich auch für die ambulante Pflichtversorgung zuständig. Dem Konzept der gemeindenahen Betreuung folgend, aber auch aufgrund der geringen Zahl stationärer Betten findet sehr viel Versorgung ambulant statt. Für die Legitimität von Zwangsbehandlungen ist ausschließlich die dringende Behandlungsbedürftigkeit eines Patienten maßgeblich, der die Behandlung ablehnt. Dabei wird kein Bezug auf die Tatbestände von Gefahren (Eigen- oder Fremdgefährdung) genommen. Eine stationäre Unterbringung ist nur möglich, wenn auch eine Behandlung stattfindet und auch in diesem Bereich gilt das Prinzip „ambulant vor stationär“, d. h. auch Zwangsbehandlungen dürfen nur dann stationär stattfinden, wenn sie ambulant nicht durchführbar sind. Die forensische Psychiatrie hat nur sehr wenige Plätze, psychisch kranke Straftäter befinden sich häufig im Strafvollzug oder belegen Betten in der Allgemeinpsychiatrie. Verglichen mit Deutschland sind weniger Betten verfügbar, die Personalausstattung insbesondere in der Pflege ist aber besser. Zwangsbehandlungen sind, bezogen auf die Einwohnerzahl, häufiger als in Deutschland, Unterbringungen (ist in Italien nur auf richterliche Anweisung möglich) und mechanische Freiheitsbeschränkungen dagegen deutlich seltener.

## Abstract (en)

Mental healthcare in South Tyrol, as everywhere in Italy, is still characterized by Law 180, which came into force in 1978 under the leadership of Franco Basaglia and Bruno Orsini. The Ministry of Health subsequently set a target number of beds of 10/100,000 inhabitants. Unlike in other parts of Italy, private clinics play a minimal role in South Tyrol. The “Psychiatric Services” are part of the state healthcare system responsible for all citizens and are also responsible for compulsory outpatient care. According to the concept of community care, also due to the small number of inpatient beds, a great deal of care is provided on an outpatient basis. Coercive measures can only be used in the case of an illness requiring urgent treatment that the patient refuses, without recourse to endangering circumstances (self-endangerment or danger to a third party). Inpatient hospitalization is only possible if treatment also takes place and the principle of “outpatient before inpatient” also applies in this context, i.e., coercive treatment can only take place as an inpatient if it cannot be carried out as an outpatient. Forensic psychiatry has very few places and mentally ill offenders are often in prison or occupy beds in general psychiatric wards. Compared to Germany there are fewer beds available but staffing levels are better, particularly for nursing. In relation to the number of inhabitants, compulsory treatment is more frequent than in Germany, whereas involuntary hospitalization and physical restraint are much rarer (only possible in Italy by court order).

## Geschichte: Die Bedeutung des Gesetzes 180 für Italien

Das „Gesetz 180 vom 13.05.1978“, gemeinhin als „Legge Basaglia“ bekannt, folgte den Vorschlägen des radikalen Psychiatriereformers Franco Basaglia (1924–1980) und des christdemokratischen Parlamentariers Bruno Orsini, ebenfalls Psychiater (* 1929). Es verbot neue Einweisungen in die in desolatem Zustand befindlichen staatlichen „manicomi“ (Irrenhäuser) und sah stattdessen vor, dass eine psychiatrische Behandlung nur an – neu zu schaffenden – kleinen psychiatrischen Abteilungen an Allgemeinkrankenhäusern möglich sein dürfe. Die Größe dieser Abteilungen wurde gesetzlich auf 15 Akutbetten beschränkt. Zugleich war die Einrichtung umfangreicher gemeindepsychiatrischer und ambulanter Behandlungsangebote vorgesehen. Jeder Psychiatrische Dienst wurde verpflichtet, in seinem Einzugsgebiet („territorio“), das zwischen 50.000 und 200.000 Einwohner umfasste, die gesamte psychiatrische Versorgung der Bevölkerung zu übernehmen.

Insbesondere wurden die Voraussetzungen einer Zwangsbegutachtung und -behandlung detailliert gesetzlich geregelt, um Patientenrechte zu schützen. Die Reform wurde innerhalb Italiens in unterschiedlichem Maße umgesetzt, wie auch in anderen Bereichen bestehen hier beträchtliche regionale Unterschiede. [1, 2]. In manchen Regionen konnte eine vorbildliche gemeindepsychiatrische Versorgung realisiert werden. In anderen blieb dies aus und die ehemaligen „manicomi“ oder andere Einrichtungen wurden als Privatkliniken, welche häufig auch vertragsgebunden mit dem öffentlichen Gesundheitsdienst zusammenarbeiten, weiterbetrieben. Private Kliniken unterschiedlicher Funktion können nämlich auch Aufgaben der staatlichen Gesundheitsversorgung übernehmen und beanspruchen damit auch einen entsprechenden Anteil des zur Verfügung stehenden Budgets. Die fundamentale Bedeutung der Reform wird daran deutlich, dass das Gesetz seit der Einführung, anders als die Psychisch-Kranken-Gesetze nahezu aller anderen europäischen Länder, bis auf den Bereich der forensischen Psychiatrie, keine relevanten Änderungen erfahren hat und in wesentlichen Grundzügen weiter von einem breiten Konsens der beteiligten Fachkräfte getragen wird.

## Regionale Besonderheiten in Südtirol

In Südtirol gab es vor Inkrafttreten des Gesetzes Nr. 180 keine psychiatrische Akutabteilung. Schwer psychisch Kranke mussten häufig in die großen psychiatrischen Kliniken der Nachbarprovinz Trient (Pergine) oder über die Staatsgrenze nach Nordtirol (Hall) gebracht werden. Nach dem verfügten Aufnahmestopp in Pergine wurden Abteilungen in Bozen (1978), Bruneck (1994), Brixen (1997) und Meran (1999) aufgebaut. Die Psychiatrie Bozen verfügt über 26 Akutbetten, Meran über 9 Betten, Brixen und Bruneck über 15 Betten für eine Bevölkerung von insgesamt 536.000 Einwohnern (davon ca. 426.000 Erwachsene). Die Bettenmessziffer fällt folglich mit 15,5/100.000 erwachsenen Einwohner etwas höher aus als im Durchschnitt Italiens. Für ganz Südtirol gibt es eine psychosomatische Klinik mit 65 Plätzen in Rodeneck (Therapiezentrum Bad Bachgart). Das Therapiezentrum wird von einem Psychologen geleitet und hat einen ganzheitlichen, integrativen Ansatz mit verhaltenstherapeutischem und systemischem Schwerpunkt. Die Struktur ist in einen psychosomatischen Bereich sowie einen Bereich für Abhängigkeitserkrankungen (v. a. Alkohol- und Medikamentenabhängigkeit, aber auch nichtsubstanzgebundene Abhängigkeiten) aufgeteilt. Die Zuweisung erfolgt über den Hausarzt oder den Facharzt für Psychiatrie und die geplanten Aufnahmen erfolgen nach einem Vorstellungsgespräch. Die Finanzierung erfolgt über das öffentliche Gesundheitssystem. Für Südtirol gibt es nur 5 forensische Plätze in Pergine in der Nachbarprovinz Trient. Private Kliniken spielen, anders als in anderen Regionen, praktisch keine Rolle. Niedergelassene Psychiater und Psychologen sind nur privat tätig und spielen im Versorgungssystem eine limitierte Rolle. Das gemeindepsychiatrische Versorgungssystem ist gut ausgebaut und umfasst 7 „Zentren für Psychische Gesundheit“, welche die ambulante und aufsuchende Versorgung garantieren, 3 Rehazentren, 1 Tageszentrum und 8 psychiatrische Wohnheime. Zusätzlich bestehen unabhängig vom Gesundheitsdienst im öffentlichen Sozialdienst angesiedelt mehrere Wohngemeinschaften und Berufstrainingszentren, Werkstätten und Treffpunkte für Menschen mit chronischen psychischen Erkrankungen.

## Gesetzliche Regelungen

In Italien besteht ein staatliches, steuerfinanziertes Gesundheitssystem. Jeder in Italien wohnhafte Bürger hat Anspruch auf Gesundheitsversorgung. Die psychiatrische Versorgung ist Teil dieses Systems. Jeder Bürger hat eine elektronische Patientenakte, die alle ärztlichen Befunde enthält und auf die er jederzeit zugreifen kann.

Die Psychiatrie in Südtirol wie in ganz Italien ist entscheidend durch die Gesetzgebung, durch das Gesetz 180 vom 13.05.1978, geprägt. Von besonderem Interesse und maßgeblich gestaltend für die Versorgung ist die Gesetzgebung bezüglich der Anwendung von Zwang. Diese nimmt keinen Bezug auf Tatbestände von Gefahren (Gefahr für den Patienten selbst oder für andere), sondern definiert als legitimierendes Merkmal für die Anwendung von Zwang ausschließlich eine „dringend behandlungsbedürftige psychische Erkrankung“ bei fehlender Behandlungsbereitschaft und fehlender Möglichkeit einer außerstationären Behandlung. Diese Merkmale sind von zwei unabhängigen Ärzten zu bestätigen, von denen einer dem öffentlichen Gesundheitssystem Italiens angehören muss. Zwangsbehandlungen sind nur dann gerechtfertigt, wenn eine schwerwiegende psychiatrische Erkrankung dringlich behandlungsbedürftig ist und andere Maßnahmen nicht ausreichen, um die Gesundheit des Patienten zu schützen sowie Schaden abzuwenden. Das Gesetz nimmt keinen expliziten Bezug auf die Einwilligungsfähigkeit, wobei durch die im Gesetz vorgegebenen Kriterien eigentlich ausschließlich einwilligungsunfähige Patienten zwangsbehandelt werden. Der Patientenwille soll, so weit wie möglich, auch im Rahmen einer Zwangsbehandlung respektiert werden. Die Anordnung erfolgt durch den Bürgermeister, welcher in Italien die höchste örtliche Instanz für das Gesundheitswesen ist, und wird vom Vormundschaftsgericht überprüft. Eine Unterbringung im Krankenhaus ist ausschließlich zum Zweck der Behandlung dieser Erkrankung möglich, eine Unterbringung ohne Behandlung ist nicht zulässig. Eine zwangsweise Begutachtung kann beim Vorliegen der genannten Voraussetzungen durch den Bürgermeister angeordnet werden. Die zulässige Dauer einer Zwangsbehandlung beträgt 7 Tage, eine Verlängerung ist mittels erneuter Antragstellung möglich.

Für die Anwendung von Zwang in der Behandlung gilt generell auch das Prinzip „ambulant vor stationär“. Zwangsbehandlungen im Krankenhaus werden als extremer Eingriff in die Freiheitsrechte angesehen. Sie dürfen nur angeordnet werden, wenn eine ambulante Behandlung (ggf. auch Zwangsbehandlung) nicht möglich oder nicht ausreichend ist und die anderen Voraussetzungen vorliegen [3]. Das heißt, auch Zwangsbehandlungen finden ambulant statt. Dies kann unter Hinzuziehung der Polizei geschehen, z. B. nach dem Verbringen des Patienten in das Ambulanzzentrum oder in die Notaufnahme der Klinik, in seltenen Einzelfällen auch zu Hause mit Öffnen der Privaträume. Im Vorfeld werden in der Regel durch den Bezugspfleger und den behandelnden Psychiater immer ambulant oder aufsuchend Bemühungen unternommen, den Patienten zu einer freiwilligen Behandlung zu motivieren. Nur wenn diese Versuche scheitern, ist eine ambulante Zwangsbegutachtung oder eine Zwangsbehandlung gerechtfertigt. Jedoch ist der territorial zuständige psychiatrische Dienst auch für solche Fälle eindeutig zuständig, selbst wenn sie bis dahin nie vom Dienst betreut wurden. Diese Regelungen sind seit 2009 landesweit einheitlich; allerdings finden in vielen Regionen tatsächlich keine ambulanten Zwangsbehandlungen statt, möglicherweise wegen des nicht unerheblichen administrativen Aufwands und weil man sich vor ambulanten Zwangsbehandlungen scheut.

## Beispiel Brixen

Die psychiatrische Abteilung befindet sich in einem an das allgemeine Krankenhaus angegliederten Gebäude mit einem großen Park und ist von der Innenstadt in wenigen Minuten zu Fuß erreichbar. Zugehörig, aber räumlich getrennt, gibt es ein ambulantes Behandlungszentrum für Allgemeinpsychiatrie (Zentrum für psychische Gesundheit) und ein Zentrum für Suchterkrankungen (im selben Gebäude) in der Innenstadt. Die Gebäude sind in einem guten Zustand, in der Krankenhausabteilung gibt es Zweibettzimmer. Alle Patientenzimmer haben Zugang auf eine große Terrasse zum Park, die Fenster sind groß, es gibt Aufenthalts- und Gruppenräume. Ein multiprofessionelles Behandlungsteam (9,75 Psychiater, 2 Assistenzärzte, 43 Pflegekräfte, 4,5 Psychologen, 4 Ergotherapeuten und 2 Sozialassistenten) versorgt die Zone mit 76.000 Einwohnern (davon 59.000 Erwachsene) stationär, ambulant, aufsuchend und mit Konsilen im Allgemeinkrankenhaus. Anders als in anderen Teilen Italiens und vielen Ländern Europas arbeiten die Fachärzte ganztags in der Klinik bzw. Ambulanz, d. h., betreiben nicht am Nachmittag eine eigene (Privat‑)Praxis. Die Abteilung verfügt über 15 stationäre Betten auf zwei Stockwerken, davon 6 in einer geschlossenen Abteilung. Zusätzlich existiert ein tagesklinischer Platz (optional auch vollstationär nutzbar). Hierbei ist zu erwähnen, dass im „Psychiatrieplan“ der Provinz Südtirol für Brixen 6 Plätze für Day- und Nighthospital vorgesehen sind, von welchen jedoch aus verwaltungstechnischen Gründen bisher nur ein Platz aktiviert werden konnte. Ebenso wurde das für den Bezirk Brixen vorgesehene Rehabilitationszentrum mit 12 Plätzen bisher aus denselben Gründen noch nicht errichtet, es befindet sich jedoch in Planung. Die Abteilung versorgt jährlich ca. 350 stationäre Fälle und ca. 2500 ambulante. Innerhalb der Klinik kommen bei erheblicher Gewalttätigkeit Fixierungen zur Anwendung, sonst auch Isolierungen. Die Überwachung erfolgt durch ein Sichtfenster im benachbarten Pflegestützpunkt, wobei bei Fixierungen, dem provinzweit geltenden Qualitätsstandard entsprechend, Kontrollen am Patienten durch das Pflegepersonal mindestens halbstündlich erfolgen müssen.

Das Zentrum für psychische Gesundheit (Ambulanz) ist architektonisch schlichter, aber funktional. Auch hier gibt es Ergotherapieräume, die ambulante Patienten regelmäßig besuchen können.

Grundsätzlich werden alle Arten psychischer Erkrankungen aufgenommen, es gibt keine Ausschlussdiagnosen. Wegen der Knappheit der Betten müssen allerdings immer wieder Priorisierungen nach Dringlichkeit und Schweregrad erfolgen. Die durchschnittliche Behandlungsdauer beträgt 17 Tage. Typische behandelte Patientengruppen sind psychotische Erkrankungen, bipolare Störungen und schwere depressive Syndrome mit Suizidalität. Aber auch Kriseninterventionen bei Borderline-Patienten und Suizidalität, die Behandlung schwerer Essstörungen sowie die Behandlung von Delir und Demenz mit Verhaltensauffälligkeiten sind möglich. Zusätzlich werden Patienten mit komplexen Abhängigkeitserkrankungen (Polytoxikomanie, komorbide psychiatrische Erkrankungen oder ausgeprägte Verhaltensauffälligkeiten) behandelt, während unkomplizierte Entzugsbehandlungen in der Medizinabteilung durchgeführt werden. Die Behandlung folgt im Wesentlichen den deutschsprachigen S3-Leitlinien. Elektrokonvulsionstherapie kommt vereinzelt zur Anwendung.

## Kennzahlen im Ländervergleich

Ländervergleiche nach ausgewählten Kennzahlen oder Indikatoren sind sehr gut geeignet, Unterschiede in den Versorgungssystemen abzubilden. Aggregierte Daten auf nationaler Ebene sind allerdings unterschiedlich verfügbar, beruhen nicht immer auf solider Datenqualität und bilden die große Varianz auf regionaler Ebene nicht ab. Wir verwenden in den vorliegenden Tabellen deshalb Daten auf nationaler oder Bundeslandebene, soweit diese aus verschiedenen Quellen verfügbar sind. Gleichzeitig ergänzen wir sie um regionale Vergleichsdaten aus einer früheren eigenen Publikation im Vergleich mit einer Klinik in Wien [4]. Die Daten aus Brixen und Bozen wurden von den beteiligten Autoren aus den vorliegenden schriftlichen Dokumenten selbst ausgewertet, dabei wurden in Brixen Daten der Jahre 2022 und 2023 gemittelt, in Bozen die Daten aus 2023 erhoben.

Die Tab. 1 zeigt Daten zu Behandlungsplätzen und personellen Ressourcen im Vergleich.

**Tab. 1:** Behandlungsplätze und personelle Ressourcen

	D gesamt oder Bundesländer	Vergleichsklinik Ravensburg	I (Brixen)	I (Bozen)
Vollstat. psychiatr. Betten/100.000 EW	70^1^	59,0^2^	25,4	10,9
Vollstat. psychosomat. Betten/100.000 EW	14^3^ 40 Reha	23,0	8,2 (in Bachgart)	8,2 (in Bachgart) + 3^4^
Tagesklinikplätze/100.000 EW	21^1^	20,0	1,7	2,5
Stationäre Behandlungen/100.000 EW	915^1^	1026	533	544,5
Teilstationäre Behandlungen/100.000 EW	199^1^	145,2	19,5	65,5
Durchschnittl. Stationsgröße (Betten)	Unbekannt	20,0	7,5	13
Durchschnittl. Verweildauer^5^	24 Tage^1^	22,1 Tage	16,8 Tage	7,95 Tage
Pflichtversorgung für Region	Überwiegend	Ja	Ja	Ja
Versorgung von Suchtkranken	Ja	Ja	Nur bei Krisen und komplexen Fällen mit eventuellen Verhaltensauffälligkeiten	Nur bei Krisen und komplexen Fällen mit eventuellen Verhaltensauffälligkeiten
Versorgung Gerontopsychiatrie	Ja, alle	Ja, alle	Ja, ohne Delir	Ja, alle
Durchschnittliche Anzahl Pflegekräften^5^ pro Schicht und Station vormittags/pro Bett	Unbekannt	3,0/0,15	2/0,27	6/0,46
Durchschnittliche Anzahl Pflegekräfte^5^ pro Schicht nachmittags/pro Bett	Unbekannt	3/0,15	2/0,27	6/0,46
Durchschnittliche Anzahl Pflegekräfte^5^ pro Schicht nachts	Unbekannt	1,9/0,095	2/0,27	6/0,46
Durchschnittliche Anzahl Ärzte pro Schicht/Station vormittags (ohne Oberarzt)	Unbekannt	2	1,5	2,5 (8.00–17.00)
Durchschnittliche Anzahl Ärzte pro Schicht/Station nachmittags (ohne Oberarzt)	Unbekannt	2	1,5	2,5
Vollzeitäquivalente angestellte Ärzte/Bett	Unbekannt	0,13^6^	0,20	0,19
Durchschnittliche Anzahl Psychologen/Bett	Unbekannt	0,04^6,7^	0,06	0,09
Ambulante Fälle/100.000 EW	Ca. 2100^1,8^	Unbekannt^9^	Ca. 4200	Ca. 4800
Psychiater/100.000 EW	18^1^	Unbekannt^9^	16	12

*EW* Einwohner

^1^ DGPPN-Dossier [5], verschiedene Quellen, Raten/100.000 EW daraus errechnet, hier analog zu Südtirol auf die volljährige Bevölkerung bezogen

^2^ Ohne Kinder- und Jugendpsychiatrie und forens. Psychiatrie

^3^ Statist. Bundesamt

^4^ Schwere Essstörungen: Einzugsgebiet Bz + Meran für 350.000 EW

^5^ Nicht differenziert nach Qualifikation

^6^ Bezogen auf vollstationäre Betten

^7^ Inkl. Psychologen i. Ausbildung u. vergleichbar

^8^ PIA + Kassenärzte, Schätzung, da Zahlen als Quartalsfälle vorliegen

^9^ Sehr unterschiedliche Tätigkeitsprofile im kassenärztlichen Bereich

Die Ergebnisse zeigen, dass in Südtirol im Vergleich zu Deutschland wesentlich weniger stationäre und vor allem teilstationäre Plätze verfügbar sind, allerdings deutlich mehr Personal pro Bett, besonders in der Pflege. In Deutschland werden deutlich mehr Patienten mit Suchterkrankungen, depressiven und Angststörungen stationär behandelt. Für eine Kerngruppe schwer erkrankter Patienten, die gerichtlich untergebracht werden müssen, zeigt Tab. 2 Indikatoren für den besonders sensiblen Bereich von Maßnahmen gegen den Willen der Betroffenen. Mit „Zwangsmaßnahmen“, soweit nicht näher spezifiziert, sind dabei Zwangsmaßnahmen irgendeiner Arzt gemeint, also Fixierung, Isolierung (wird nicht praktiziert) oder Zwangsbehandlung, mit oder ohne Anwendung körperlichen Zwangs. Der Anteil unfreiwilliger Patienten ist (trotz der niedrigen Bettenzahl) vergleichbar mit Deutschland. Fixierung und Isolierung sind in den italienischen Vergleichsabteilungen etwas seltener als in Deutschland, bevölkerungsbezogen deutlich seltener; Zwangsbehandlung ist dagegen häufiger, weil Unterbringung nur zur Behandlung möglich ist. Insofern sind alle untergebrachten Patienten auch von Zwangsmaßnahmen betroffen. Ambulante Zwangsbehandlung, gesetzlich sogar priorisiert, findet statt, beschränkt sich aber dennoch auf wenige Fälle im Jahr. Gerichtlich angeordnete Behandlungsweisungen (Community Treatment Orders [CTO] im internationalen Schrifttum) sind in Einzelfällen möglich, z. B. im Zusammenhang mit Sorgerechtsfragen.

**Tab. 2:** Anwendung von Zwang

	D gesamt oder Bundesländer	Vergleichsklinik Ravensburg^1^	I (Brixen)	I (Bozen)
% unfreiwillige Aufenthalte (unterschiedl. Unterbringungsarten)	Unbekannt	9,8 %	9,8 %	5,9 %
% Aufnahmen von Fixierung oder Isolierung betroffen	8,0%^2^	7,3 %	6,2 %	3,9 %
% von Zwangsmaßnahmen betroffen/% unfreiwillig	Unbekannt	0,74	0,63	0,66
Unterbringungen/100.000 EW	168^3^	154,8	33,1^5^	32,1^5^
Pat. mit Zwangsmaßnahmen im Krankenhaus/100.000 EW	69^3^	87,6	33,1^5^	32,1
% Aufnahmen mit Zwangsbehandlung	0,4 %^4^	1,0 %	9,8 %^5^	5,9 %^5^
Zwangsbehandlungen/100.000 EW	8,2^4^	11,8	33,1^5^	32,1^5^
Ambulante Zwangsbehandlung	Nicht zulässig	Nicht zulässig	3/Jahr 5,1/100.000 EW	–
Aggressive Übergriffe	Unbekannt	20/100 Aufnahmen	Unbekannt	10/100 Aufnahmen
% Öffnung der Stationen (8.00–20.00)	Unbekannt	64,7 %	60 %	100 %
Forensisch-psychiatrische Plätze	15/100.000 EW^3^		1,19/100.000 EW (5 Plätze insgesamt verfügbar)	1,19/100.000 EW (5 Plätze insgesamt verfügbar)

^1^ Aus Steinert und Scharfetter [4]

^2^ Adorjan et al. [6]

^3^ DGPPN-Dossier [5], verschiedene Quellen, Raten/100.000 EW daraus errechnet

^4^ Flammer et al. [7]

^5^ Als Unterbringungen wurden die angeordneten Zwangsbehandlungen gezählt, Unterbringungen i. e. S. gibt es nicht

## Forensische Psychiatrie

In der forensischen Psychiatrie Pergine, in der Nachbarprovinz Trient, stehen für Südtirol insgesamt lediglich 5 materiell und personell gut ausgestattete Behandlungsplätze zur Verfügung. Verglichen mit Deutschland ist die bevölkerungsbezogene Zahl sehr viel geringer.

## Probleme

Mit einer Bettenkennzahl von 15,5 psychiatrischen Akutbetten pro 100.000 Einwohnern ist Südtirol zwar über dem vom Staat geforderten Standard von 10 Betten pro 100.000, besitzt aber im Verhältnis zu Deutschland deutlich weniger stationäre und teilstationäre Behandlungsplätze. Die von Basaglia angestoßene Entwicklung einer intensiven territorialen Betreuung hat dazu geführt, dass sich die Ressourcen im ambulanten Bereich konzentrieren müssen und die Zentren für Psychische Gesundheit multidisziplinär intensiv und auch aufsuchend tätig werden. Deshalb bestehen konstant Versorgungsschwierigkeiten für psychisch Kranke, die längerer stationärer oder teilstationärer Aufenthalte bedürfen. Dies geht häufig zulasten der betroffenen Familien [8]. Die Beschränkung der Bettenzahl an den italienischen psychiatrischen Abteilungen lässt keine Ausdifferenzierung in spezialisierte Abteilungen zu, worunter häufig die stationäre Versorgung von Suchtpatienten und gerontopsychiatrischen Patienten leidet. Die Psychiatriereform hatte bis zum Jahr 2008 die forensische Psychiatrie nicht miteingeschlossen. Bis 2008 gab es im gesamten Staatsgebiet 6 Haftanstalten für psychisch kranke Rechtsbrecher mit 1639 Behandlungsplätzen. Dem Basaglia-Prinzip getreu wurde in der Folge eine Territorialisierung auch der forensisch psychiatrischen Einrichtungen eingeleitet. Bis 2017 lösten 30 kleine regionale Zentren (REMS) mit nur 607 Behandlungsplätzen die früheren Anstalten ab. In diese therapeutisch-rehabilitative Einrichtungen sollen nach Gerichtsbeschluss die nicht oder nur teilweise schuldfähigen psychisch kranken Rechtsbrecher mit hoher sozialer Gefährlichkeit aufgenommen werden; die Aufenthaltsdauer darf das zeitliche Strafmaß nicht überschreiten. Jenen mit geringer sozialer Gefährlichkeit werden personenbezogene, nichtfreiheitsentziehende Alternativmaßnahmen auferlegt und sie werden über die psychiatrischen Dienste territorial betreut. Die gutgemeinte Reform führte zu einem deutlichen Mangel an Unterbringungsplätzen, sodass Anfang 2024 ca. 700 Verurteilte auf eine Aufnahme in eine REMS warteten [9]. Daraus folgt, dass eine unbekannte Zahl psychisch erkrankter Straftäter in Haftanstalten platziert ist oder sich mit damit einhergehenden Risiken in Freiheit befindet. Auch gibt es längerdauernde Fehlplatzierungen in den wenigen Behandlungsplätzen der allgemeinpsychiatrischen Abteilungen.

## Diskussion

Der Vergleich muss vor dem Hintergrund der Tatsache beurteilt werden, dass Italien für die staatliche Gesundheitsversorgung nur 9,2 % des Bruttoinlandsprodukts ausgibt, diese Werte liegen unter dem europäischen Durchschnitt. Deutschland liegt mit 12,9 % an der Spitze der EU-Länder [10]. Auch der Anteil der Ausgaben für psychische Gesundheit ist in Deutschland deutlich höher, wenngleich die Quellenlage hier inkonsistent ist. Gemessen daran bietet das staatliche Gesundheitswesen in Italien eine bemerkenswert effektive Versorgung mit einer eindeutigen Priorisierung ambulanter Behandlung auch schwer erkrankter Menschen. Bei deutlich niedrigeren stationären Bettenmessziffern sind ambulante Behandlungen (wenngleich die Vergleichbarkeit nur bedingt gegeben ist) tendenziell sogar häufiger (dies auch bereits seit Jahrzehnten [11]), sektorübergreifende Behandlung ist dabei vollständig realisiert. Möglicherweise wird die Versorgung dadurch erleichtert, dass in Italien seit Langem deutlich niedrigere Prävalenzraten für psychische Erkrankungen als anderswo in Europa vorliegen. Im Vergleich von 16 untersuchten Staaten waren sie teilweise halb so hoch wie der europäische Durchschnitt [12]. In gewisser Weise bildet sich dieser Umstand auch in den Suizidraten ab, die zu den niedrigsten in Europa zählen [13]. Südtirol hingegen hat innerhalb Italiens eine auffallend hohe Suizidrate, die jener Deutschlands nahekommt und durchschnittlich fast doppelt so hoch ist wie die gesamtitalienische [13, 14]. Seit Jahrzehnten bekannt und wohl auch berechtigt ist die Kritik, dass die Knappheit verfügbarer Betten (auch in der forensischen Psychiatrie) dazu führt, dass längerfristige stationäre Behandlung mit rehabilitativer Zielsetzung zu selten durchgeführt werden und dies häufig zulasten der Angehörigen geht [2, 8, 15]. Eine Untersuchung in 30 zufällig ausgewählten Versorgungseinheiten Italiens ergab massive familiäre Belastungsmomente: 97 % der Angehörigen schizophren Erkrankter fühlten sich alleingelassen, 83 % depressiv, 73 % hatten ihre Hobbys aufgegeben und 68 % hatten keinen Urlaub mehr gemacht [15]. Spezialisierungen und Differenzierungen in der stationären Behandlung sind nur sehr eingeschränkt möglich, was wahrscheinlich ein Nebeneffekt der strengen Sektorisierung ist. Bei sehr knappem Bettenangebot in Kleinstabteilungen müssen Patienten verschiedenster Diagnosen gemeinsam behandelt werden. Bemerkenswert aus deutscher Sicht sind die Regelungen für Zwangsbehandlungen. Das weitgehend unveränderte „Basaglia-Gesetz“ von 1978, das von den Fachkräften weiterhin als sehr funktional angesehen wird, kann diesbezüglich rückblickend als „großer Wurf“ eingeschätzt werden. Es ist primär medizinisch konzipiert und knüpft die Unterbringung zur Behandlung an eine dringend notwendige und vom Patienten verweigerte Behandlung, nicht aber an Gefahrentatbestände. Das UN-Komitee zur Umsetzung der Behindertenrechtskonvention beurteilt dies als eine kritikwürdige Zugrundelegung eines „medizinischen Modells“ [16]. Auch aus medizinethischer Sicht könnte kritisiert werden, dass das Kriterium der fehlenden Einwilligungsfähigkeit fehlt, wobei die vorgegebenen Kriterien für eine Zwangsbehandlung diese quasi implizieren. Dennoch sind Unterbringungen und freiheitsbeschränkende Zwangsmaßnahmen bevölkerungsbezogen deutlich weniger häufig als in Deutschland. Dies gilt auch unter Einbeziehung der in Deutschland sehr kritisch diskutierten ambulanten Zwangsbehandlung. Zwangsbehandlungen, die konsequent anstelle einer Unterbringungsmaßnahme durchzuführen sind, sind allerdings – sowohl in der Klinik als auch bevölkerungsbezogen – häufiger als in Deutschland, wo diesbezüglich relativ hohe Hürden bestehen [17]. Ein kausaler Zusammenhang zwischen häufigeren Zwangsbehandlungen und weniger Anwendung von Zwang insgesamt kann vermutet werden, ist aber mittels derartiger epidemiologischer Daten nicht zu belegen. Wir sind der Ansicht, dass die Analyse der Versorgung im südlichsten Teil des deutschen Sprachraums, in Südtirol, interessante Anregungen für die künftig notwendige Transformation der psychiatrischen Versorgung in Deutschland geben kann.

## Data Availability

Die verwendeten Originaldaten mit der Errechnung der Kenngrößen sind über die Autoren erhältlich.
